# A novel nonsense mutation in *CRYBB1* associated with autosomal dominant congenital cataract

**Published:** 2008-04-18

**Authors:** Juhua Yang, Yihua Zhu, Feng Gu, Xiang He, Zongfu Cao, Xuexi Li, Yi Tong, Xu Ma

**Affiliations:** 1 Biomedical Engineering Center of Fujian Medical University, Fuzhou, Fujian, China; 2 Department of Genetics, National Research Institute for Family Planning, Beijing, China; 3 Department of Ophthalmology, First Affiliated Hospital of Fujian Medical University, Fuzhou, Fujian, China; 4 Peking Union Medical College, Beijing, China; 5 Ophthalmic Center, 180 Hospital of PLA, Quanzhou, Fujian, China

## Abstract

**Purpose:**

To identify the molecular defect underlying an autosomal dominant congenital nuclear cataract in a Chinese family.

**Methods:**

Twenty-two members of a three-generation pedigree were recruited, clinical examinations were performed, and genomic DNA was extracted from peripheral blood leukocytes. All members were genotyped with polymorphic microsatellite markers adjacent to each of the known cataract-related genes. Linkage analysis was performed after genotyping. Candidate genes were screened for mutation using direct sequencing. Individuals were screened for presence of a mutation by restriction fragment length polymorphism (RFLP) analysis.

**Results:**

Linkage analysis identified a maximum LOD score of 3.31 (recombination fraction [θ]=0.0) with marker D22S1167 on chromosome 22, which flanks the β-crystallin gene cluster (*CRYBB3*, *CRYBB2*, *CRYBB1*, and *CRYBA4*). Sequencing the coding regions and the flanking intronic sequences of these four candidate genes identified a novel, heterozygous C→T transition in exon 6 of *CRYBB1* in the affected individuals of the family. This single nucleotide change introduced a novel BfaI site and was predicted to result in a nonsense mutation at codon 223 that changed a phylogenetically conserved amino acid to a stop codon (p.Q223X). RFLP analysis confirmed that this mutation co-segregated with the disease phenotype in all available family members and was not found in 100 normal unrelated individuals from the same ethnic background.

**Conclusions:**

This study has identified a novel nonsense mutation in *CRYBB1* (p.Q223X) associated with autosomal dominant congenital nuclear cataract.

## Introduction

Cataract is the most common treatable cause of visual disability in both childhood (congenital cataract) and in adults (age-related cataract). The prevalence estimates for congenital cataract vary over 10-fold from 0.6/10,000 to 6.0/10,000, depending on the method of ascertainment. The condition can occur in isolation or as part of a more complex syndrome [1]. Over the past few years, remarkable progress has been made toward our understanding of the process of cataractogenesis. Currently, there are more than 40 genetic loci to which isolated or primary cataracts have been mapped and more than 30 genes have been characterized, although this number is constantly increasing [2].

Crystallins are known to constitute about 90% of the water-soluble proteins of the lens and contribute to transparency and refractive properties due to a uniform concentration gradient [3,4]. They fall into two classes, the α-crystallin family and the β/γ-crystallin superfamily. Because of high levels of expression in the lens, crystallins represent compelling candidate genes for inherited cataracts. Indeed, defects in crystallin genes have been shown to be associated with human cataract formation [4]. To date, many mutations in 12 human crystallin genes have been associated with inherited autosomal dominant (AD) and/or autosomal recessive (AR) cataract. These genes are linked to 1q for *CRYZ* [5], 2q for *CRYGC/D* [6,7], 3q for *CRYGS* [8], 11q for *CRYAB* [9], 17q for *CRYBA1/3* [10-16], 21q for *CRYAA* [17], and 22q for *CRYBB1* [18-21], *CRYBB2* [22-27], *CRYBB3* [28], and *CRYBA4* [29].

CRYBB1 is a major subunit of the β-crystallins and comprises 9% of the total soluble crystallin in the human lens [30]. The amino- and carboxyl-terminal extensions of β-crystallins are presumed to be of importance in protein aggregation and orientation, and loss of the terminal arms can either increase or decrease dimerization of the β-crystallins, which causes cataract formation [31]. Mackay et al. [18] identified a mutation in *CRYBB1*, G220X, to be associated with autosomal dominant pulverulent cataract and suggested that the mutation disrupts the fourth Greek key motif, probably causing instability of CRYBB1. Three additional mutations in *CRYBB1* associated with AD and/or AR congenital cataract have previously been reported [19-21].

Herein, we report the identification of a novel nonsense mutation (c.C737T) in *CRYBB1* that introduces a translation stop codon at Gln (p.Q223X). This mutation is responsible for autosomal dominant congenital nuclear cataract affecting a three-generation Chinese family. To our knowledge, this is the first reported case of nuclear cataracts associated with the *CRYBB1* mutation Gln223X.

## Methods

### Clinical evaluations and DNA specimens

**Figure 1:**
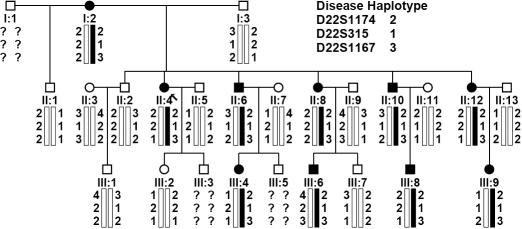
Cataract pedigree and haplotype analysis. Pedigree and haplotype analysis of the cataract family shows the segregation of three microsatellite markers on chromosome 22q11.2–12.1, which is listed in descending order from the centromere. Squares and circles symbolize males and females, respectively. The clear and shaded symbols denote unaffected and affected individuals, respectively. The proband is marked with an arrow.

**Figure 2:**
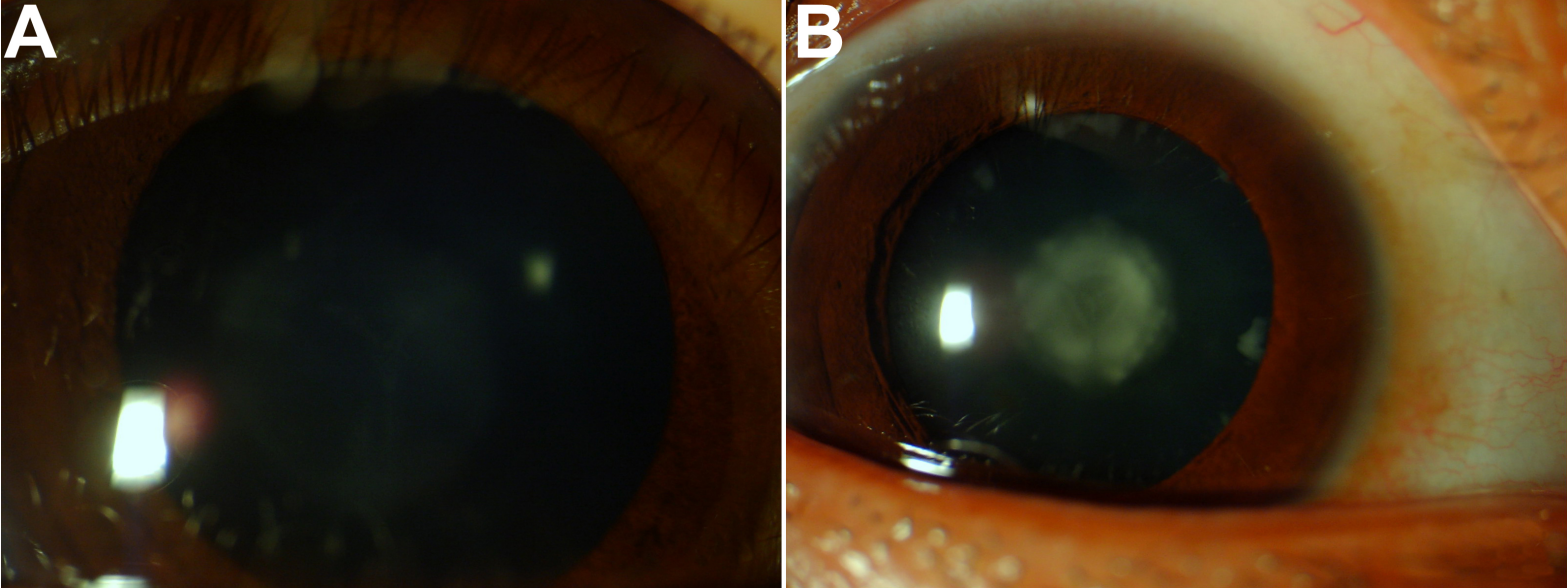
Photograph of affected individuals. All of the affected family members exhibited a nuclear cataract phenotype. The lens opacity increased with age in the affected individuals of this family (**A**: III:9, aged 5; **B**: II:4, aged 41).

A three-generation family with non-syndromic congenital nuclear cataracts was recruited at the First Affiliated Hospital of Fujian Medical University, Fuzhou, China. Twenty-two individuals took part in this study including 10 affected and 12 unaffected individuals (Figure 1). Clinical and ophthalmologic examinations were performed on the affected individuals as well as on the unaffected family members. The diagnosis of cataract was confirmed in each affected patient by ophthalmologic examination. Phenotype was documented by slit lamp photography (Figure 2). Informed consent was obtained from each participant, consistent with the Declaration of Helsinki. Genomic DNA was extracted from peripheral blood leukocytes using the Wizard Genomic DNA Purification Kit (Promega, Beijing, China) according to manufacturer’s instructions.

### Genotyping

Exclusion analysis was performed in affected individuals from different generations with polymorphic microsatellite markers flanking 32 candidate genes [20] to determine whether all affected individuals share the same allele. The genotyping was performed using microsatellite markers as previously described [21,32]. Pedigree and haplotype data were managed using Cyrillic (version 2.1) software.

### Linkage analysis

Two-point linkage LOD scores (Z) were calculated as previously described [21,32]. The marker order and distances between the markers were taken from the NCBI and GDB databases.

### Mutational analysis

Genomic DNA samples from affected and unaffected members of the family and from 100 ethnically matched control individuals were screened for mutations in *CRYBB3*, *CRYBB2*, *CRYBB1*, and *CRYBA4* with a combination of direct cycle sequencing and restriction fragment length polymorphism (RFLP) analysis. Briefly, individual exons of these four candidate genes were amplified by polymerase chain reaction (PCR), and PCR products were sequenced on an ABI 3730XL Automated Sequencer (PE Biosystems, Foster City, CA), using gene specific primers, as previously described [21].

For RFLP analysis, specific primers were designed to amplify part of *CRYBB1* exon 6, forward: 5′-TAG AGC CTG GTG ACT TCC G-3′, and reverse: 5′-GGT AGC AGA GTG AGG TGT GG-3′. The reaction was performed with PCR master mix (Premix *Taq*^TM^, *Ex Taq* Version; TAKARA Biotechnology Co., Ltd, Dalian, China), and conditions were as follows: 94 °C for 1 min; 35 cycles at 94 °C for 30 s, 58 °C for 30 s, and 72 °C for 30 s; and 1 cycle of 72 °C for 7 min. Restriction enzyme digestion was performed using BfaI (TaKaRa) according to manufacturer’s instructions.

Amino acid sequences for CRYBB1 were retrieved from NCBI. Multiple sequence alignments of CRYBB1 from various species were performed using DNAMAN software (version 5.0, Lynnon Corp., Quebec, Canada).

## Results

### Clinical data

This three-generation family included 10 affected individuals with congenital nuclear cataract and 15 unaffected individuals (Figure 1). All affected individuals presented disc-like opacities in the central nucleus region of both lenses. However, younger affected individuals (III:4, III:6, III:8, and III:9; aged between 5 and 12) presented thin, gray opacities located mainly in the fetal nuclei and less in the embryonic nuclei. The older affected individuals (I:2, II:4, II:6, II:8, II:10, and II:12; aged between 31 and 65) showed dense, white opacities distributed throughout the embryonic and fetal nuclei. Overall, lens opacity increased with age in the affected individuals of this family (Figure 2A,B). Ophthalmic records confirmed that the opacities were present from birth and that there was no family history of other ocular or systemic abnormalities.

**Table 1:** Two-point LOD scores for linkage between cataract locus and chromosome 22 markers.

**Marker**	**LOD scores at recombination for values of θ**							
	**0**	**0.01**	**0.05**	**0.1**	**0.2**	**0.3**	**0.4**	**Zmax**
D22S1174	0.6	0.59	0.56	0.51	0.41	0.29	0.16	0.6
D22S315	0.9	0.88	0.77	0.64	0.38	0.16	0.03	0.9
D22S1167	3.31	3.26	3.04	2.76	2.15	1.46	0.69	3.31

Two-point LOD scores for linkage in microsatellite markers across the β-crystallin gene cluster in the chromosomal regions 22q11.2-q12.1 is displayed. Significant linkage was found with marker D22S1167 (Z_max_=3.31, at θ=0.0).

### Linkage and haplotype analysis

All known cataract-related candidate genes were excluded by allele-sharing analysis except for a cluster of β-crystallin genes on chromosome 22 (*CRYBB3*, *CRYBB2*, *CRYBB1*, and *CRYBA4*; data not shown). Significant and suggestive linkage was found with markers D22S1167 (LOD score [Z]=3.31 at recombination fraction [θ]=0.0), D22S315 (LOD score [Z]=0.903, recombination fraction [θ]=0.0), and D22S1174 (LOD score [Z]=0.602, recombination fraction [θ]=0.0; Table 1). These markers closely flank the *CRYBB3*, *CRYBB2*, *CRYBB1*, and *CRYBA4* gene cluster. Haplotype analysis indicated that the disease gene was close to these three markers (Figure 1), implying that one or more of the geness, *CRYBB3*, *CRYBB2*, *CRYBB1*, and *CRYBA4*, might be responsible for the disease.

**Figure 3:**
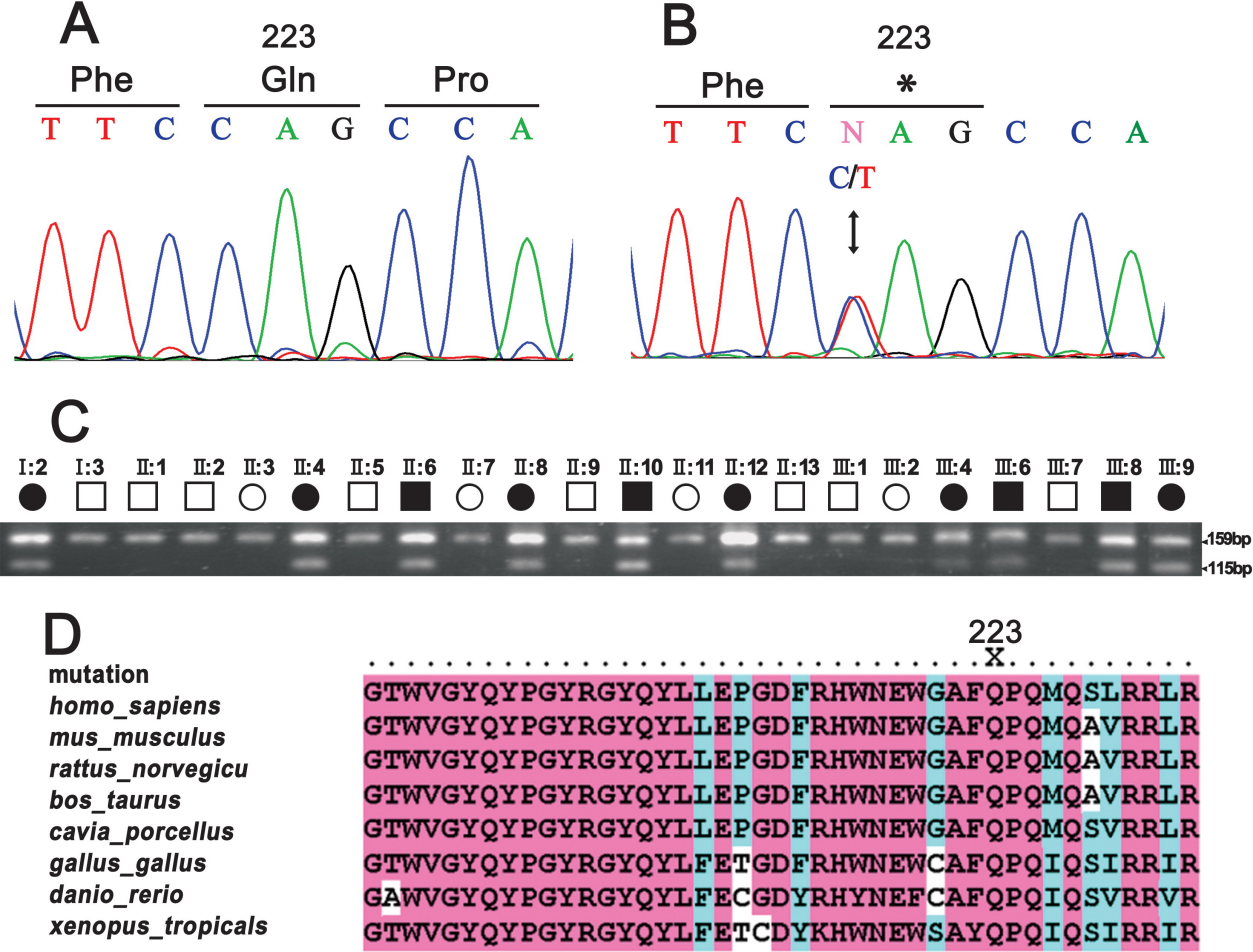
Mutation analysis of *CRYBB1*. **A**: The sequence chromatogram of a wild type allele shows glutamine (CAG) at codon 223. **B**: The sequence chromatogram of a mutant allele shows a heterozygous C→T transition that changed glutamine 223 to a stop codon (TAG). **C**: RFLP analysis illustrates that the mutation, Q223X, introduces a new BfaI site. This mutation-specific BfaI digestion pattern co-segregated with the disease phenotype. Squares and circles symbolize males and females, respectively. The clear and shaded symbols denote unaffected and affected individuals, respectively. **D**: Multiple sequence alignment of the fourth Greek key motif of CRYBB1 is shown from *Homo sapiens* (codons 191–233), *Mus musculus*, *Rattus norvegicus*, *Bos Taurus*, *Cavia porcellus*, *Gallus gallus*, *Danio rerio*, and *Xenopus tropicalis*. The Gln223 residue is highly conserved. “X” indicates premature chain-termination mutations in human CRYBB1 (Q223X).

### Mutation analysis

Direct sequencing of the coding regions and of the flanking intronic sequences of *CRYBB3*, *CRYBB2*, *CRYBB1*, and *CRYBA4* in two affected individuals revealed no nucleotide changes except a heterozygous c.C737T transition in exon 6 of *CRYBB1* (nucleotide change based on sequence NM_001887; gi:21536279; Figure 3B). This single nucleotide change introduced a novel BfaI site, C/TAG, and was predicted to result in a nonsense or chain-termination mutation at codon 223. This changed a phylogenetically conserved glutamine to a stop codon (p.Q223X). The remainder of the *CRYBB1* coding sequence showed no change. BfaI digestion analysis confirmed that this sequence alteration, Q223X, co-segregated with affected individuals but not with unaffected family members (Figure 3C). In addition, this single-nucleotide change was not detected in 100 normal unrelated individuals from the same ethnic background. This suggested that it was the causative mutation rather than a rare polymorphism in strong linkage disequilibrium with the disease in this pedigree. The multiple sequence alignments generated using DNAMAN software showed that the Gln at position 223 of human CRYBB1 is highly conserved in *Mus musculus*, *Rattus norvegicus*, *Bos taurus*, *Cavia porcellus*, *Gallus gallus*, *Danio rerio*, and *Xenopus tropicalis* (Figure 3D).

## Discussion

The β-crystallins belong to the β/γ-crystallin superfamily, which contain central globular cores, consisting of Greek key motifs, a term used because the motif contains a quadruple repeat of a β-sheet that resembles the characteristic pattern found on classical Greek pottery [33]. Many studies have demonstrated that mutations in β-crystallin genes, including *CRYBB1* [18-21], *CRYBB2* [22-27], *CRYBB3* [28], *CRYBA1/3* [10-16], and *CRYBA4* [29], are associated with inherited AD and/or AR cataract.

In the present study, we have demonstrated that an autosomal dominant congenital nuclear cataract condition is caused by a mutation in *CRYBB1*. This mutation resulted in a nonsense or chain-termination mutation at codon 223 in the Greek key IV motif. This changed a glutamine to a stop codon (p.Q223X) and was predicted to truncate wild type CRYBB1 by 30 amino acids. Mackay et al. [7] have identified a similar chain-termination mutation, G220X, in *CRYBB1* that is associated with autosomal dominant pulverulent cataract. This mutation also occurs in the Greek key IV motif and was predicted to truncate wild type CRYBB1 by 33 amino acids. Furthermore, the expression of recombinant human CRYBB1 in bacteria showed that the truncated G220X mutant was significantly less soluble than the wild type, suggesting that this mutation disrupted the fourth Greek key motif, which resulted in the instability of the molecule [18].

In addition, another similar chain-termination mutation (Q155X) in *CRYBB2* [22,23] was predicted to remove the last 51 amino acids of CRYBB2, deleting ∼90% of the fourth Greek-key motif (codons 155–192) and the entire COOH-terminal region (codons 193–205). The Q155X mutant in CRYBB2 shows decreased ordered structure and stability, but the partially unfolded protein retains some dimer structure, suggesting this truncation mutation might contribute to cataract formation [34]. Cohen et al. [20] describe a recessively inherited congenital cataract condition caused by a homozygous delG168 mutation in exon 2 of *CRYBB1* that generates a frameshift, leading to a missense protein sequence at amino acid 57 and truncation at amino acid 107 of the 252-amino acid CRYBB1. From the above evidence, we suggest that the Q223X mutant, lacking more than 25% of the fourth Greek-key motif and the entire COOH-terminal region (Figure 3D), has decreased stability or has altered higher order aggregation, which results in cataractogenesis. Interestingly, Willoughby et al. [19] report another dominant mutation in *CRYBB1* associated with autosomal dominant congenital cataract and micro-cornea. This mutation generated an X253R change, leading to the elongation of the COOH-terminus. This study also implies that *CRYBB1* plays a role not only in cataractogenesis but also in ocular development [19].

All reported mutations of *CRYBB1* associated with autosomal dominant congenital cataract occur in exon 6, which encodes the Greek key IV and the COOH-terminal arm [18,19,21]. Although the deletion of COOH-terminal residues from rat CRYBB2 [35], chicken CRYBB1 [36], and human CRYBB1 [37] did not significantly impair solubility or the ability to form dimers in vitro, all known mutations (such as G220X CRYBB1 [18], Q223X CRYBB1 (in this study), Q155X CRYBB2 [22,23] and del185QSVR188 CRYBB2 [38]) suggest that disruption of the fourth Greek key motif in CRYBB1 or in CRYBB2 results in β-crystallin instability and/or cataract disease.

In conclusion, we report a novel nonsense mutation (Q223X) in *CRYBB1* in a family with autosomal dominant congenital nuclear cataract. It occurs within the same gene, *CRYBB1*, but the clinical phenotypes were dissimilar with those reported by Mackay et al. [18], Willoughby et al. [19], and Cohen et al. [20], and were similar with our previously described phenotype [21]. These results provide strong evidence that *CRYBB1* is a pathogenic gene for congenital cataract and the Greek key motif IV is an important structural domain in CRYBB1 for cataract formation.

## References

[1] Reddy MA, Francis PJ, Berry V, Bhattacharya SS, Moore AT (2004). Molecular genetic basis of inherited cataract and associated phenotypes.. Surv Ophthalmol.

[2] Shiels A, Hejtmancik JF (2007). Genetic origins of cataract.. Arch Ophthalmol.

[3] Wistow GJ, Piatigorsky J (1988). Lens crystallins: the evolution and expression of proteins for a highly specialized tissue.. Annu Rev Biochem.

[4] Graw J (1997). The crystallins: genes, proteins and diseases.. Biol Chem.

[5] Heinzmann C, Kojis TL, Gonzalez P, Rao PV (1994). Zigler JSJr, Polymeropoulos MH, Klisak I, Sparkes RS, Mohandas T, Bateman JB. Assignment of the zeta-crystallin gene (CRYZ) to human chromosome 1p22-p31 and identification of restriction fragment length polymorphisms.. Genomics.

[6] Heon E, Priston M, Schorderet DF, Billingsley GD, Girard PO, Lubsen N, Munier FL (1999). The gamma-crystallins and human cataracts: a puzzle made clearer.. Am J Hum Genet.

[7] Stephan DA, Gillanders E, Vanderveen D, Freas-Lutz D, Wistow G, Baxevanis AD, Robbins CM, VanAuken A, Quesenberry MI, Bailey-Wilson J, Juo SH, Trent JM, Smith L, Brownstein MJ (1999). Progressive juvenile-onset punctate cataracts caused by mutation of the gammaD-crystallin gene.. Proc Natl Acad Sci USA.

[8] Sun H, Ma Z, Li Y, Liu B, Li Z, Ding X, Gao Y, Ma W, Tang X, Li X, Shen Y (2005). Gamma-S crystallin gene (CRYGS) mutation causes dominant progressive cortical cataract in humans.. J Med Genet.

[9] Berry V, Francis P, Reddy MA, Collyer D, Vithana E, MacKay I, Dawson G, Carey AH, Moore A, Bhattacharya SS, Quinlan RA (2001). Alpha-B crystallin gene (CRYAB) mutation causes dominant congenital posterior polar cataract in humans.. Am J Hum Genet.

[10] Kannabiran C, Rogan PK, Olmos L, Basti S, Rao GN, Kaiser-Kupfer M, Hejtmancik JF (1998). Autosomal dominant zonular cataract with sutural opacities is associated with a splice mutation in the betaA3/A1-crystallin gene.. Mol Vis.

[11] Bateman JB, Geyer DD, Flodman P, Johannes M, Sikela J, Walter N, Moreira AT, Clancy K, Spence MA (2000). A new betaA1-crystallin splice junction mutation in autosomal dominant cataract.. Invest Ophthalmol Vis Sci.

[12] Qi YH, Jia HY, Huang SZ, Lin H, Gu JZ, Su H, Zhang TY, Gao Y (2003). Autosomal dominant congenital nuclear cataract caused by a deletion mutation in the beta A1-crystallin gene.. Zhonghua Yi Xue Yi Chuan Xue Za Zhi..

[13] Qi Y, Jia H, Huang S, Lin H, Gu J, Su H, Zhang T, Gao Y, Qu L, Li D, Li Y (2004). A deletion mutation in the betaA1/A3 crystallin gene (CRYBA1/A3) is associated with autosomal dominant congenital nuclear cataract in a Chinese family.. Hum Genet.

[14] Reddy MA, Bateman OA, Chakarova C, Ferris J, Berry V, Lomas E, Sarra R, Smith MA, Moore AT, Bhattacharya SS, Slingsby C (2004). Characterization of the G91del CRYBA1/3-crystallin protein: a cause of human inherited cataract.. Hum Mol Genet.

[15] Ferrini W, Schorderet DF, Othenin-Girard P, Uffer S, Héon E, Munier FL (2004). CRYBA3/A1 gene mutation associated with suture-sparing autosomal dominant congenital nuclear cataract: a novel phenotype.. Invest Ophthalmol Vis Sci.

[16] Lu S, Zhao C, Jiao H, Kere J, Tang X, Zhao F, Zhang X, Zhao K, Larsson C (2007). Two Chinese families with pulverulent congenital cataracts and deltaG91 CRYBA1 mutations.. Mol Vis.

[17] Litt M, Kramer P, LaMorticella DM, Murphey W, Lovrien EW, Weleber RG (1998). Autosomal dominant congenital cataract associated with a missense mutation in the human alpha crystallin gene CRYAA.. Hum Mol Genet.

[18] Mackay DS, Boskovska OB, Knopf HL, Lampi KJ, Shiels A (2002). A nonsense mutation in CRYBB1 associated with autosomal dominant cataract linked to human chromosome 22q.. Am J Hum Genet.

[19] Willoughby CE, Shafiq A, Ferrini W, Chan LL, Billingsley G, Priston M, Mok C, Chandna A, Kaye S, Héon E (2005). CRYBB1 mutation associated with congenital cataract and microcornea.. Mol Vis.

[20] Cohen D, Bar-Yosef U, Levy J, Gradstein L, Belfair N, Ofir R, Joshua S, Lifshitz T, Carmi R, Birk OS (2007). Homozygous CRYBB1 deletion mutation underlies autosomal recessive congenital cataract.. Invest Ophthalmol Vis Sci.

[21] Wang J, Ma X, Gu F, Liu NP, Hao XL, Wang KJ, Wang NL, Zhu SQ (2007). A missense mutation S228P in the CRYBB1 gene causes autosomal dominant congenital cataract.. Chin Med J (Engl).

[22] Litt M, Carrero-Valenzuela R, LaMorticella DM, Schultz DW, Mitchell TN, Kramer P, Maumenee IH (1997). Autosomal dominant cerulean cataract is associated with a chain termination mutation in the human beta-crystallin gene CRYBB2.. Hum Mol Genet.

[23] Gill D, Klose R, Munier FL, McFadden M, Priston M, Billingsley G, Ducrey N, Schorderet DF, Héon E (2000). Genetic heterogeneity of the Coppock-like cataract: a mutation in CRYBB2 on chromosome 22q11.2.. Invest Ophthalmol Vis Sci.

[24] Santhiya ST, Manisastry SM, Rawlley D, Malathi R, Anishetty S, Gopinath PM, Vijayalakshmi P, Namperumalsamy P, Adamski J, Graw J (2004). Mutation analysis of congenital cataracts in Indian families: identification of SNPS and a new causative allele in CRYBB2 gene.. Invest Ophthalmol Vis Sci.

[25] Yao K, Tang X, Shentu X, Wang K, Rao H, Xia K (2005). Progressive polymorphic congenital cataract caused by a CRYBB2 mutation in a Chinese family.. Mol Vis.

[26] Bateman JB, von-Bischhoffshaunsen FR, Richter L, Flodman P, Burch D, Spence MA (2007). Gene conversion mutation in crystallin, beta-B2 (CRYBB2) in a Chilean family with autosomal dominant cataract.. Ophthalmology.

[27] Pauli S, Söker T, Klopp N, Illig T, Engel W, Graw J (2007). Mutation analysis in a German family identified a new cataract-causing allele in the CRYBB2 gene.. Mol Vis.

[28] Riazuddin SA, Yasmeen A, Yao W, Sergeev YV, Zhang Q, Zulfiqar F, Riaz A, Riazuddin S, Hejtmancik JF (2005). Mutations in betaB3-crystallin associated with autosomal recessive cataract in two Pakistani families.. Invest Ophthalmol Vis Sci.

[29] Billingsley G, Santhiya ST, Paterson AD, Ogata K, Wodak S, Hosseini SM, Manisastry SM, Vijayalakshmi P, Gopinath PM, Graw J, Héon E (2006). CRYBA4, a novel human cataract gene, is also involved in microphthalmia.. Am J Hum Genet.

[30] Lampi KJ, Ma Z, Shih M, Shearer TR, Smith JB, Smith DL, David LL (1997). Sequence analysis of betaA3, betaB3, and betaA4 crystallins completes the identification of the major proteins in young human lens.. J Biol Chem.

[31] Berbers GA, Boerman OC, Bloemendal H, de Jong WW (1982). Primary gene products of bovine beta-crystallin and reassociation behavior of its aggregates.. Eur J Biochem.

[32] Gu F, Zhai H, Li D, Zhao L, Li C, Huang S, Ma X (2007). A novel mutation in major intrinsic protein of the lens gene (MIP) underlies autosomal dominant cataract in a Chinese family.. Mol Vis.

[33] Blundell T, Lindley P, Miller L, Moss D, Slingsby C, Tickle I, Turnell B, Wistow G (1981). The molecular structure and stability of the eye lens: x-ray analysis of gammacrystallin II.. Nature.

[34] Liu BF, Liang JJ (2005). Interaction and biophysical properties of human lens Q155* betaB2-crystallin mutant.. Mol Vis.

[35] Trinkl S, Glockshuber R, Jaenicke R (1994). Dimerization of bB2-crystallin: the role of the linker peptide and the N- and C-terminal extensions.. Protein Sci.

[36] Coop A, Goode D, Sumner I, Crabbe MJ (1998). Effects of controlled mutations on the N- and C-terminal extensions of chick lens bB1 crystallin.. Graefes Arch Clin Exp Ophthalmol.

[37] Bateman OA, Lubsen NH, Slingsby C (2001). Association behaviour of human bB1-crystallin and its truncated forms.. Exp Eye Res.

[38] Chambers C, Russell P (1991). Deletion mutation in an eye lens b-crystallin.. J Biol Chem.

